# History of medication-assisted treatment and its association with initiating others into injection drug use in San Diego, CA

**DOI:** 10.1186/s13011-017-0126-1

**Published:** 2017-10-03

**Authors:** Maria Luisa Mittal, Devesh Vashishtha, Shelly Sun, Sonia Jain, Jazmine Cuevas-Mota, Richard Garfein, Steffanie A. Strathdee, Dan Werb

**Affiliations:** 1 Division of Infectious Diseases and Global Public Health, Department of Medicine, University of California San Diego, 9500 Gilman Drive, La Jolla, CA 92093-0507 USA; 2 Division of Biostatistics and Bioinformatics, Department of Family Medicine and Public Health, University of California San Diego, 9500 Gilman Drive, La Jolla, CA 92093-0622 USA

**Keywords:** Opioid substitution therapy, HIV prevention, HCV prevention, People who inject drugs, Methadone, Opioid agonist treatment, Injection initiation assistance

## Abstract

**Background:**

Medication-assisted treatment (MAT) remains the gold standard for the treatment of opioid use disorder. MAT also reduces the frequency of injecting among people who inject drugs (PWID). Relatedly, data suggest that PWID play a key role in the initiation of others into drug injecting by exposing injecting practices to injection-naïve drug users. Our primary objective was to test whether a history of MAT enrollment is associated with a reduced odds of PWID providing injection initiation assistance.

**Methods:**

*Preventing Injecting by Modifying Existing Responses* (PRIMER; NIDA DP2-DA040256–01), is a multi-site cohort study assessing the impact of socio-structural factors on the risk that PWID provide injection initiation assistance. Data were drawn from a participating cohort of PWID in San Diego, CA. The primary outcome was reporting ever providing injection initiation assistance; the primary predictor was reporting ever being enrolled in MAT. Logistic regression was used to model associations between MAT enrollment and ever initiating others into injecting while adjusting for potential confounders.

**Results:**

Participants (*n* = 354) were predominantly male (*n* = 249, 70%). Thirty-eight percent (*n* = 135) of participants reported ever initiating others into injection drug use. In multivariate analysis, participants who reported a history of MAT enrollment had significantly decreased odds of ever providing injection initiation assistance (Adjusted Odds Ratio [AOR]: 0.62, 95% Confidence Interval [CI]: 0.39–0.99).

**Conclusions:**

These preliminary findings suggest an association between MAT enrollment and a lower odds that male PWID report providing injection initiation assistance to injection-naïve drug users. Further research is needed to identify the pathways by which MAT enrollment may impact the risk that PWID initiate others into drug injecting.

## Background

Medication-assisted treatment (MAT) remains the gold standard of biomedical care for opioid use disorder, and is effective in reducing the frequency of injecting among people who inject drugs (PWID) [1–4]. This is important given the growing scientific consensus that PWID play a key role in the expansion of injection-related epidemics by exposing and directly initiating injection-naïve persons into injection drug use (IDU) [5, 6]. With an estimated 12 million PWID worldwide, and an increasing prevalence of opioid-related morbidity and mortality in North America and elsewhere, the prevention of opioid IDU initiation (e.g. heroin) has major public health implications [1, 2, 7, 8]. This is particularly the case as the period immediately following IDU initiation has been shown to be associated with a higher risk of HIV and HCV acquisition [9].

MAT includes opioid agonist treatment (i.e., methadone; also known as opioid substitution therapy), in combination with counseling and behavioral therapies to treat opioid use disorder [1–4]. Given that MAT is associated with reductions in the frequency of opioid injecting among PWID as well as street-based injecting in particular, we hypothesize that MAT enrollment may have a secondary preventive impact on the risk that PWID expose and initiate others into injecting [3–10]. This study therefore investigated the potential association between a history of MAT enrollment and reporting injection initiation assistance among PWID.

## Methods

### Study design

*Preventing Injecting by Modifying Existing Responses* (PRIMER; NIDA DP2-DA040256–01), is a multi-site study pooling data from cohort studies of PWID in four countries (San Diego, USA; Tijuana, Mexico; Vancouver, Canada; and Paris, Marseille, Bordeaux and Strasbourg, France) to assess the impact of socio-structural factors on the risk that PWID initiate others into injection [5]. For the present analysis, data were drawn from a cohort study of PWID in San Diego, California (*Study of Tuberculosis, AIDS, and Hepatitis C Risk* [STAHR] II; NIDA R01DA031074). To be eligible, participants had to be ≥18 years old, report last IDU ≤ 30 days prior to baseline enrollment.

### Participants and measures

Participants completed an interviewer-administered questionnaire assessing sociodemographics, IDU practices, and enrollment in health services including MAT (i.e., methadone) at baseline and at four semiannual follow-up visits. Specific questions related to providing injection initiation assistance were introduced as part of the PRIMER study at the 24-month follow-up wave (i.e., August 2014). Participants provided written informed consent. This study was approved by the University of California San Diego Human Research Protection Program.

Twenty-four month follow-up data were employed in cross-sectional analyses because PRIMER study questions were anchored at this visit. The primary outcome was reporting ever initiating others into IDU, (i.e., reporting having “ever helped someone inject who had never injected before”). The primary predictor of interest was a history of MAT enrollment defined as at least one report of MAT enrollment during any study visit.

### Statistical analyses

Fisher’s exact test was used to evaluate univariate associations between ever initiating others into IDU and the independent variables. Multivariate logistic regression analysis was performed to determine whether reporting ever initiating others was associated with ever being enrolled in MAT, independent of potential confounders such as age, gender, and years since first injection. We also studied interactions between gender and MAT enrollment in a separate multivariate model, given previous data suggesting gender differences in injection initiation risk behaviors [6, 11–15]. Statistical analyses were performed in R version 3.1.1 (http://www.r-project.org). The likelihood ratio statistic (LRS) was used to compare nested models; LRS with *p* < 0.05 were used to determine whether a variable should be retained in the model.

## Results

Participants (*n* = 354) were predominantly male (*n* = 249, 70%), with a mean age of 47 years (Interquartile Range [IQR]: 38–55), and a median of 24 years of IDU (IQR: 13–35). Thirty-eight percent of participants (*n* = 135) reported ever providing injection initiation assistance, and 39% (*n* = 137) reported ever having been enrolled in MAT. The proportion of MAT enrollment for males was 67.9% (*n* = 93) and 32.1% (*n* = 44) for female and transgender participants. The majority of participants (*n* = 304, 86%) reported having ever injected heroin.

As shown in Table 1, there was a significantly higher proportion of participants ≤30 years old who reported ever providing injection initiation assistance compared with older participants aged 31–50 and ≥51 years (69.0% vs. 37.7% vs. 32.9%, Fisher’s exact *p* < 0.01).

**Table 1:** Univariate analysis of factors potentially associated with ever providing injection initiation assistance among persons who inject drugs in San Diego, CA (*n* = 354)

Variable	Did not ever initiate others into injection (*n* = 219)	Ever initiated others into injection (*n* = 135)	P-value^a^
Age			
≤ 30	9 (31.0%)	20 (69.0%)	0.001
31–50	104 (62.3%)	63 (37.7%)	
≥ 51	106 (67.1%)	52 (32.9%)	
Gender, *n* = 352^b^			
Female	64 (65.3%)	34 (34.7%)	0.460
Transgender	2 (40.0%)	3 (60.0%)	
Male	153 (61.5%)	96 (38.6%)	
Marital Status			
Married	28 (68.3%)	13 (31.7%)	0.398
Other	191 (61.0%)	122 (39.0%)	
Ever been in prison			
No	103 (60.2%)	68 (39.8%)	0.585
Yes	116 (63.4%)	67 (36.6%)	
Years since first injection, *n* = 353^b^			
≤ 5 years	10 (50.0%)	10 (50.0%)	0.504
6–10 years	30 (61.2%)	19 (38.8%)	
> 10 years	179 (63.0%)	105 (37.0%)	
Ever injected heroin			
No	31 (62.0%)	19 (38.0%)	>0.999
Yes	188 (61.8%)	116 (38.2%)	
Ever injected cocaine			
No	56 (62.9%)	33 (37.1%)	0.900
Yes	163 (61.5%)	102 (38.5%)	
Ever injected meth			
No	29 (76.3%)	9 (23.7%)	0.054
Yes	190 (60.1%)	126 (39.9%)	
Ever enrolled in MAT			
No	128 (59.0%)	89 (41.0%)	0.178
Yes	91 (66.4%)	46 (33.6%)	

^a^Fisher’s exact test; ^b^Change in sample size due to different number of observations available for each variable; *MAT* Medication-assisted Treatment

As shown in Table 2, each year increase in age was associated with a decreased odds of ever providing injection initiation assistance (Wald χ^2^ = 13.27, degrees of freedom [*df*] = 1, *p* < 0.01), while reporting a higher number of years since first injection was associated with an increased odds initiating others (Wald χ^2^ = 5.53, *df* = 1, *p* = 0.02). PWID reporting a history of MAT enrollment had significantly decreased odds of initiating others into injecting (Wald χ^2^ = 4.04, *df* = 1, *p* = 0.04; see Table 2). Additionally, the inclusion of an interaction term in a separate multivariate model did not significantly impact the association between gender and a history of MAT enrollment (χ^2^ = 1.13, *df* = 1, *p* = 0.29; data not shown).

**Table 2:** Multivariate Logistic Regression to assess factors associated with ever providing injection initiation assistance in San Diego, CA

Variable	AOR	95% CI	P-value (*df*)	Test Statistic^a^
Age	0.94	0.91–0.97	<0.01 (1)	13.27
Years since first injecting	1.04	1.00–1.07	0.02 (1)	5.53
Male gender	1.18	0.72–1.92	0.52 (1)	0.41
Ever enrolled in MAT	0.62	0.39–0.99	0.04 (1)	4.04

^a^Wald test in the multivariate logistic regression model; *AOR* Adjusted Odds Ratio, *95% CI* 95% Confidence Interval, *df* degrees of freedom, *MAT* Medication-assisted Treatment

## Discussion

Along with its effectiveness in supporting the management of opioid use disorder [1–4], these preliminary results suggest that MAT enrollment may also be associated with a reduced risk that PWID initiate others into IDU. Specifically, results suggest that among PWID participants, a history of MAT enrollment was associated with a 38% reduction in the odds of having reported initiating others into IDU. This suggests a need to further explore potential pathways by which MAT enrollment may influence the risk that PWID provide injection initiation assistance.

In line with other studies, we also found that each year increase in age was associated with a decreased risk of providing injection initiation assistance [16, 17]. However, in contrast to these studies we observed an association between a higher number of years since first injection and an increased risk of providing injection initiation assistance.

Multiple studies have reported on gender differences in injection initiation, including data suggesting that men are most often initiated by men comparted to women [12–15, 18]. Additionally, data suggest that some gender-responsive programs may influence the capacity of PWID to engage with supplementary health services offered during MAT enrollment [11, 19–25]. However, the effect of a history of MAT enrollment on providing injection initiation assistance did not differ significantly between male and female participants in our sample. Further quantitative and qualitative studies are needed to more clearly delineate potential differences by gender with respect to injection initiation risk and uptake of MAT.

To our knowledge, this is the first study to investigate the potential impact of MAT enrollment on providing injection initiation assistance. As such, and due to the exploratory nature of this analysis, results should be interpreted cautiously. First, survey items assessing lifetime initiation of others into IDU were limited to the final follow-up of a 24-month observational cohort study, and we were therefore unable to identify the temporal ordering of the dependent and independent variables, and, as such, cannot confirm the direction of the causal association. It may be the case that both enrollment in MAT and avoiding the initiation of others into IDU are both proxy markers of increased capacity by participants to manage their opioid use and we note that this will be the subject of future longitudinal study from our group. Second, providing injection initiation assistance is a highly stigmatized behavior and likely resulted in under-reporting of this behavior [13, 26]. However, there is no reason to believe that differential under-reporting occurred among PWID based on MAT enrollment history; thus, the effect of this bias is likely to be toward a null finding. Despite these limitations, this study provides preliminary evidence particular to opioid users of an association between MAT enrollment and the provision of injection initiation assistance that should be investigated in longitudinal study.

## Conclusions

Given the harms associated with recent increases in opioid use across North America [1, 4], this study highlights the need to further investigate the potential impact of MAT as a preventive intervention to reduce not only the incidence of negative injection-related health outcomes experienced by opioid users but also incident cases of IDU initiation.

## Abbreviations

AIDS: Acquired Immune Deficiency Syndrome
AOR: Adjusted Odds Ratio
CA: California
CI: Confidence Interval
DF: Degrees of Freedom
HCV: Hepatitis C Virus
HIV: Human Immunodeficiency Virus
IDU: Injection Drug Use
IQR: Interquartile Range
LRS: Likelihood Ratio Statistic
MAT: Medication-assisted treatment
NIDA: National Institute of Drug Abuse
PRIMER: Preventing Injecting by Modifying Existing Responses Study
PWID: People who Inject Drugs
USA: United States of America
